# Extraction of Pyrrole from Its Mixture with *n*-Hexadecane Using Ionic Liquids and Their Binary Mixtures

**DOI:** 10.3390/molecules28248129

**Published:** 2023-12-16

**Authors:** Sorfina Amran, Muhammad Zulhaziman Mat Salleh, Hanee F. Hizaddin, Abdullah Amru Indera Luthfi, Ahmad Alhadid, Mohamed Kamel Hadj-Kali

**Affiliations:** 1 Department of Chemical and Process Engineering, Faculty of Engineering and Built Environment, Universiti Kebangsaan Malaysia, Bangi 43600, Malaysia; 2 University Malaya Centre for Ionic Liquids (UMCiL), University of Malaya, Kuala Lumpur 50603, Malaysia; hanee@um.edu.my; 3 Biothermodynamics, TUM School of Life Sciences, Technical University of Munich, Maximus-von-Imhof-Forum 2, D-85354 Freising, Germany; ahmad.alhadid@tum.de; 4 Chemical Engineering Department, College of Engineering, King Saud University, P.O. Box 800, Riyadh 11421, Saudi Arabia

**Keywords:** denitrification, ionic liquids, liquid–liquid extraction, protic ionic liquid, NRTL, COSMO-RS

## Abstract

The conventional hydrodenitrogenation method is expensive and involves the use of catalysts and harsh procedures. In the last few years, ionic liquids (ILs) have gained attention as a promising alternative solvent for fuel oil extractive denitrogenation. In this work, the Conductor-like Screening Model for Real Solvents (COSMO-RS) was used to screen 173 potential ILs as solvents for fuel oil. Two ILs (1-ethyl-3-methylimidazolium dicyanamide ([EMIM][N(CN)_2_]) and 1-ethyl-3-methylimidazolium methanesulfonate ([EMIM][MeSO_3_])) were selected for experimental investigation. The experimental liquid–liquid extraction of pyrrole (taken as the model nitrogen compound) from *n*-hexadecane (the model fuel) was conducted at 298 K and 1 atm with feed concentrations of pyrrole ranging from 10 to 50 wt%, using either the two pure ILs or their mixtures with dimethylformamide or ethylene glycol. Moreover, the NRTL model was effectively used to correlate the experimental tie lines. This work shows that the use of a binary mixture of ILs with a conventional solvent results in good selectivity, but has a low capacity for extracting pyrrole compounds. On the other hand, using an IL-IL mixture exhibits good results for both capacity and selectivity. All the ternary systems tested showed positive slopes, indicating that the nitrogen compounds had a higher affinity for the IL and binary mixture extract phase. In fact, the extraction efficiency for all the systems shows promising results. This characteristic is advantageous, as it requires less solvent to remove nitrogen compounds.

## 1. Introduction

The fuel processing industry considers denitrogenation to be a crucial step, because the existence of nitrogen-containing aromatic compounds can disrupt the standard hydrodesulfurization process by causing competitive adsorption and poisoning of the catalyst [1]. In addition, the release of nitrogen oxides into the air during combustion leads to air pollution and the formation of acid rain. As a result, numerous nations have implemented strict guidelines to restrict nitrogen levels in fuels. For instance, the Euro 6 standards, introduced in September 2014, set a strict NOx limit of 80 mg/km and 60 mg/km for diesel and petrol cars, respectively [2]. Hydrodenitrogenation (HDN) is the conventional process for removing nitrogen compounds from fuel. However, this process is an expensive catalytic process requiring a high hydrogen supply and a high temperature (600 K) and pressure (300 atm) [3]. Nitrogen compounds found in fuel oil can be classified into two main categories: basic compounds, characterized by five-membered pyrrolic ring systems, and non-basic compounds, characterized by six-membered pyridinic ring systems [4]. The HDN process is ineffective for eliminating persistent aromatic nitrogen compounds, such as pyridine, pyrrole, and their derivatives.

The liquid–liquid extraction (LLE) process, especially extractive denitrogenation (EDN), is an appealing alternative due to its ability to operate under ambient conditions. Nonetheless, the meticulous choice of extracting solvents is crucial for this process. Numerous investigations have explored this procedure, employing conventional organic solvents, such as methanol [5], ethanol [6], *N*-methylformamide [7], *N*,*N*-dimethylformamide [8], and 1-methyl-2-pyrrolidinone [9]. Even though these solvents have exhibited a high level of extractive ability in their respective ternary systems, their use on a larger scale could be impeded by their solvent properties, particularly their high volatility. Ionic liquids (ILs) are gaining increasing recognition as a versatile class of customizable solvents with a wide range of applications, owing to the unique and boundless permutations of their anion and cation arrangements. In addition, ILs are perceived as environmentally friendly solvents due to their minimal vapor pressure. In fact, to regenerate ionic liquids, the volatile components can be eliminated via evaporation under high vacuum conditions [10]. Numerous researchers have explored the efficacy of various types of ILs for carrying out desulfurization [11,12,13,14,15], denitrification [16,17,18] and dearomatization [19,20,21] processes on both model solutions and actual refinery streams.

One of the primary concerns with using ILs as solvents on an industrial scale is their cost and complex synthesis procedures. Moreover, as many ILs are available with different properties, the experimental investigation of each IL or IL mixture is impossible. Thus, it is necessary to establish a systematic approach with which to preselect the top ILs with a high capacity and selectivity for EDN. The Conductor-like Screening Model for Real Solvents (COSMO-RS) [22] can predict the activity coefficient of the components in liquid mixtures, employing only their molecular structure [23,24,25,26] In our previous study, the COSMO-RS was employed to screen ILs for extracting nitrogen compounds from *n*-hexadecane as a model fuel, using imidazolium and pyridinium-based ILs. The results showed that the COSMO-RS predictions for the ternary tie lines corresponded well with the experimental outcomes (with a root-mean-square deviation (RMSD) of less than 5%) [27].

Acknowledging the lack of experimental ternary liquid–liquid equilibrium (LLE) data regarding the use of ILs and binary mixtures for extracting nitrogen compounds, this work involved three phases: (1) the computational screening of suitable ILs, (2) experimental validation, and (3) an evaluation of the ILs mixing performance with quaternary systems to enhance the separation efficiency. In phase (1), the COSMO-RS was used to systematically screen the potential ILs in the separating heterocyclic nitrogen compound, i.e., pyrrole, by predicting their values of infinite dilution capacity and selectivity. Next, in phase (2), the performance of the selected ILs was validated experimentally through the acquisition of liquid–liquid extraction (LLE) data and the analysis of their extractive performance. In phase (3), based on the computational screening and experimental validations, the extractive performance was validated using three binary mixtures of ILs.

## 2. Results and Discussion

### 2.1. Computational Screening of ILs

**Figure 1:**
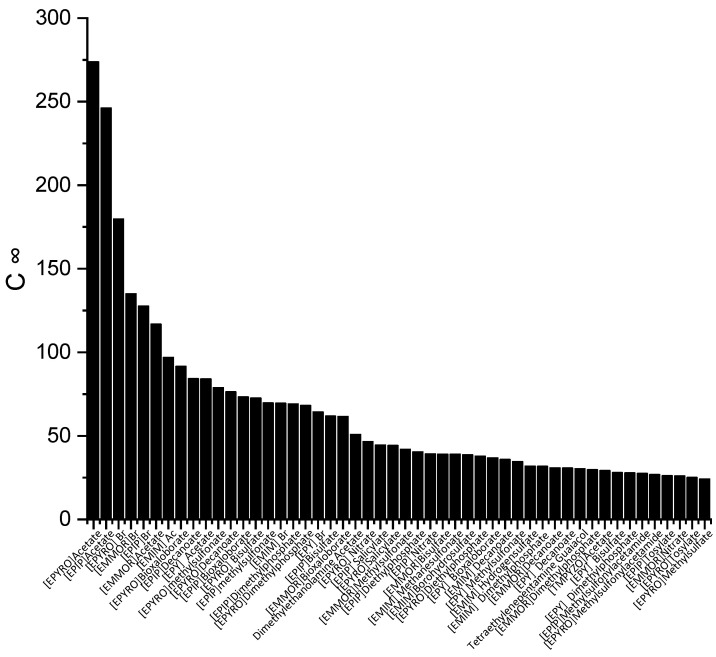
The top 50 ILs with high capacity from 173 studied ILs at 298 K.

The screening process for the potential solvents involved the evaluation of two criteria: capacity and selectivity at infinite dilution. A higher selectivity suggests improved separation and a reduced number of extraction stages, whereas a higher capacity indicates a larger amount of extraction. In the COSMO-RS, the maximum amount of solute that a solvent can dissolve can be represented by the capacity at infinite dilution (*C^∞^*), which is expressed using Equation (6). Figure 1 illustrates the top 50 ILs with respect to the *C^∞^* at a temperature of 298 K.

**Figure 2:**
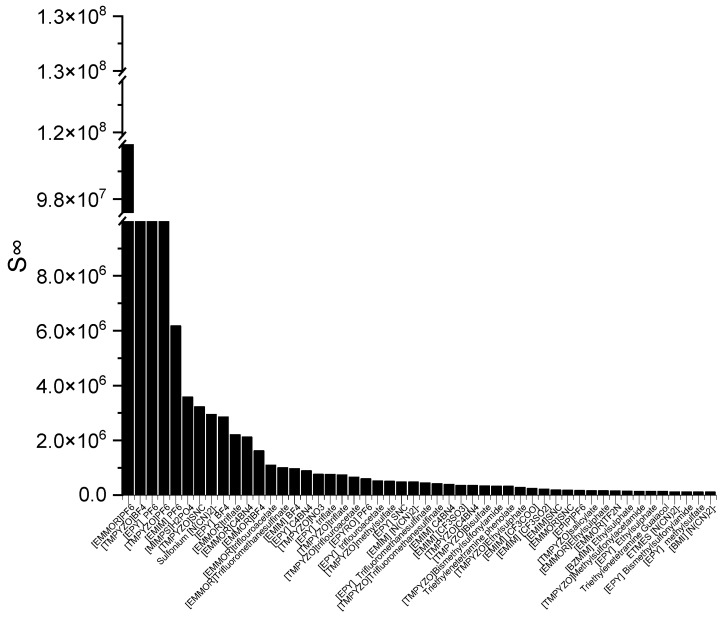
The top 50 ILs with high selectivity from 173 studied ILs at 298 K.

The ILs in Figure 1 are ranked from the largest to smallest *C^∞^* values. As observed, the *C^∞^* is affected by the type of cation family, in the order of EPYRO > EPIP > EMMOR > EPY > EMIM > TMPYZO. This trend can be explained due to the lesser amount of steric hindrance of the first three compared to EMIM, TMPYZO, and EPY, which are encompassed by an aromatic ring. Furthermore, Figure 1 also implies that anions with lesser numbers of heteroatomic atoms, such as acetate, bromide, and decanoate, result in a significant effect in terms of capacity. A lesser negative charge reduces the coulombic force and increases the solvent capacity. Figure 2 presents the top 50 ILs based on the calculated *S^∞^* at 298 K.

The corresponding ILs are listed from the largest to smaller order of their COSMO-RS reading result. The cation family was shown to have a significant influence on the *S^∞^*. The cation families EMMOR and TMPYZO showed the greatest influence on the *S^∞^*, followed by EPY and EMIM. This could be caused by the influence of the heteroatom in EMMOR, i.e., N and O, which increased the charge of the cation. In addition, the presence of an aromatic ring in TMPYZO, which is rich in electron density, resulted in a strong π-π interaction with the pyrrole compound.

In a liquid–liquid extraction, an ideal IL would be one possessing high capacity and selectivity. However, the actual extraction process typically found that low selectivity resulted in high capacity, and vice versa. While the capacity determines the flow rate of the circulating solvent and, consequently, the size of the reactor, it is also a crucial factor in the solvent selection process. The selectivity needs to be properly assessed as well. As a result, there ought to be criteria that can consider both qualities. The performance index (PI) is one of the techniques used to complement the inverse proportionality between capacity and selectivity.

**Figure 3:**
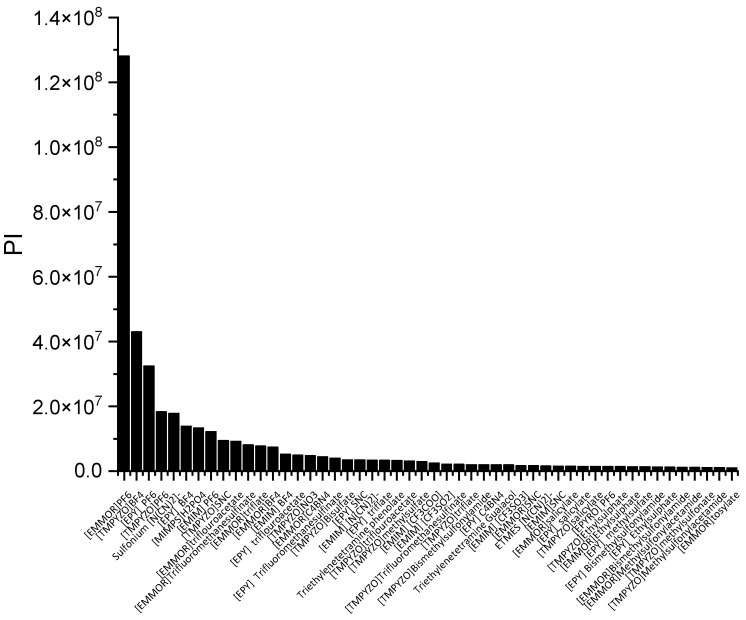
The top 50 ILs with high PI from 173 studied ILs at 298 K.

In Figure 3, [EMMOR][PF_6_] is shown to be the best candidate among the other ILs studied. However, for its application on an industrial scale, the cost and toxicity of the IL must be considered. For instance, the presence of a halide anion may result in equipment corrosiveness [28] In this work, we considered the IL ranking after the screening, and we selected two relatively cheaper ILs that fell into the categories of high capacity, [EMIM][MeSO_3_], and high selectivity, [EMIM][N(CN)_2_)]. In addition, dimethyl formamide (DMF) and ethylene glycol (EG) were chosen to represent conventional solvents with high capacity and selectivity, respectively. Table 1 summarizes the top 10 ILs based on the PI.

#### 2.1.1. Effect of Cation Alkyl Chain Length

**Figure 4:**
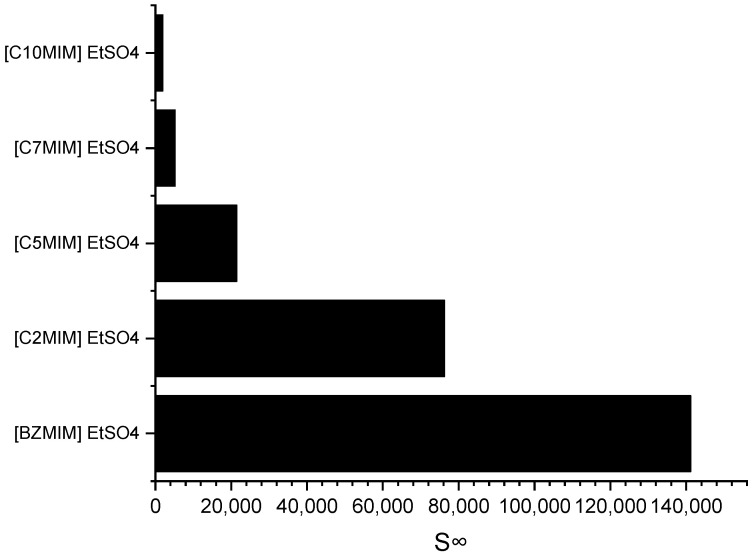
Effect of the alkyl chain length of imidazolium-based ILs cations on selectivity at infinite dilution.

Imidazolium cations are widely used in the field of ILs application due to their ability to exhibit tuneable properties, such as miscibility, melting point, and viscosity. Consequently, an imidazolium-based cation was selected for investigating the impact of the alkyl chain length on selectivity. Figure 4 depicts the effect of the alkyl chain length on selectivity, as determined through the COSMO-RS screening predictions.

It can be observed that as the alkyl chain length increases, the selectivity at infinite dilution decreases in the following order: C_10_MIM < C_7_MIM < C_5_MIM < C_2_MIM < BZMIM. The observed decrease in selectivity can be attributed to the reduced accommodation capacity of the pyrrole molecules, which arises from the increased steric hindrance on the imidazolium ring. However, interestingly, the results for BZMIM show a deviation from this trend. The presence of the benzyl group in BZMIM is expected to enhance selectivity due to its electron-rich nature and the potential for stronger π-π interactions between the aromatic and pyrrole compounds, facilitated by an additional π system. This stronger interaction is anticipated because the aromatic ring possesses an extended electron ring system, further contributing to the unique behavior of BZMIM in this study [29].

The COSMO-RS prediction results are consistent with those of a prior study by Ferreira et al. (2012). This source reported a decrease in the predicted aromatic selectivity as the alkyl chain length of the cation increased, specifically for *n*-hexane-benzene-[cation][TF2N] systems with imidazolium-based cations, and the order of selectivity was [EMIM] > [BMIM] > [OMIM] > [DMIM] > [C_12_MIM] [30]. In general, ILs that exhibit high selectivity have low capacity. In this case, the ILs with a BZMIM cation exhibited the lowest capacity at infinite dilution, as shown in Table 2. This can be attributed to the presence of a benzyl group, which renders the cation more cationic, meaning a higher positive charge, thereby increasing the coulombic force between the cation and anion. This, in turn, weakens the ability of the IL to accommodate the pyrrole compound at infinite dilution [29].

#### 2.1.2. Effect of Anions

Increasing the COSMO volume of the anions enhances the selectivity of the ILs for nitrogen species in general [31]. The COSMO volume quantifies the spatial extent or size of the anion in the IL. It provides information about the anion’s ability to interact with the surrounding cations and the other species within the IL. However, the observed trend differs, as the increase is dependent on the number of heteroatoms present in most anions, such as fluorine, sulfur, nitrogen, and oxygen [32]. Another aspect of the anion that can be quantified is the presence of double bonds or triple bonds like thiocyanate, or the size of the anion or the alkyl chain length of the anion, which plays an important role in determining selectivity and capacity. Thus, in this screening, [EMIM][PF_6_]_,_ [EMIM][SNC], [EMIM][C_7_H_7_O_3_S] (tosylate), [EMIM][Br], and [EMIM][C_10_H1_9_O_2_] (decanoate), were selected to study the effect of the anion on the *S^∞^* and *C^∞^*. Table 3 represents the chosen ILs, based on the COSMO-RS screening, used to study the effect of the anion.

Based on Table 3, the *S^∞^* increases in the order of [EMIM][PF_6_] > [EMIM][SNC] > [EMIM][C_7_H_7_O_3_S] > [EMIM][C_10_H_19_O_2_] > [EMIM][Br]. As stated earlier, the presence of heteroatoms influences selectivity, with [EMIM][PF_6_] exhibiting the highest selectivity, followed by [SCN]. This can be explained by the fact that higher selectivity is typically associated with a number of heteroatoms. The lowest selectivity at *S^∞^* was observed with the halide anion [Br]^−^, due to its lower number of heteroatoms, resulting in a lower charge value. Regarding the *C^∞,^* [EMIM][Br] and [EMIM][C_10_H_19_O_2_] (decanoate) displayed a higher *C^∞^* compared to the other ILs. The compactness of the cation and anion in [EMIM][Br] results in less steric hindrance, allowing for the easier accommodation of the pyrrole compound. Conversely, with [EMIM][C_10_H_19_O_2_], as the length of the anion increases, the coulombic and inductive forces weaken, leading to a loss of stacking structure and the easier accommodation of the pyrrole compound. Further studies are required to support this assertion by calculating the interaction energy. A lower interaction energy between the cation and anion would weaken their interaction and increase the free volume effect [33].

#### 2.1.3. Effect of Cations

The interaction between cations is mainly facilitated through N (heteroaromatic)-H (cation) hydrogen bonds [34], where N and H refer to the atoms present in the nitrogen compound and the cation, respectively. The interaction is also facilitated by a CH (cation)-π (nitrogen species) interaction [35]. In this section, six cation families, namely, imidazolium (EMIM), pyrrolidinium (EPYRO), piperidinium (EPIP), morpholinium (EMMOR), pyridinium (EPY), and pyrazolinium (TMPYZO), with the same anion [Br]^−^, will be discussed in general to determine the effect of the cation family. Table 4 presents the ionic liquids with different types of cation families to see the effect on *S^∞^* and *C^∞^*.

Based on Table 4, the *S^∞^* increases in the order of TMPYZO > EPY > EMIM > EPIP > EMMOR > EPYRO. This is due to the aromatic rings in the imidazolium, pyridinium, and pyrazolium cations having π-electron systems, which exhibit increased selectivity towards pyrrole [29]. In fact, for TMPYZO, the methyl groups in TMPYZO can alter the electronic properties of the pyrazolium ring. They can induce local electronic effects that influence the electrostatic interactions between TMPYZO and pyrrole. These electronic effects can enhance the selectivity of TMPYZO towards pyrrole by promoting favorable interactions, and reducing interactions with other molecules that do not possess compatible electronic characteristics. In summary, a notable trade-off exists between capacity and selectivity in ionic liquids (ILs). Those with high capacity typically demonstrate low selectivity, while ILs with low capacity often exhibit high selectivity. This trend is commonly observed in solvent screening studies for extraction. Thus, the performance index, PI, is pivotal to obtain both criteria (capacity + selectivity).

#### 2.1.4. Effect of Protic Ionic Liquids on Capacity and Selectivity

This section provides a brief discussion of the impact of protic ionic liquids (PILs) on the *S^∞^* and *C^∞^*, considering their distinct characteristics compared to aprotic ionic liquids (APILs). PILs are formed through the transfer of protons from a Brønsted acid to a Brønsted base, enabling the formation of hydrogen bonding [36]. While PILs have shown promising results in gas absorption studies, particularly in H_2_S removal, their application to EDN remains limited. Therefore, this section investigates the effects of five different PILs, namely, triethylenetetramine phenolate ([TETA][Phe]), triethylenetetramine guaiacol ([TETA][Guac]), tetraethylenepentamine phenolate ([TEPA][Phe]), tetraethylenepentamine guaiacol ([TEPA][Guac]), and dimethylethanolamine acetate ([DMEA][Ac]), on EDN.

**Figure 5:**
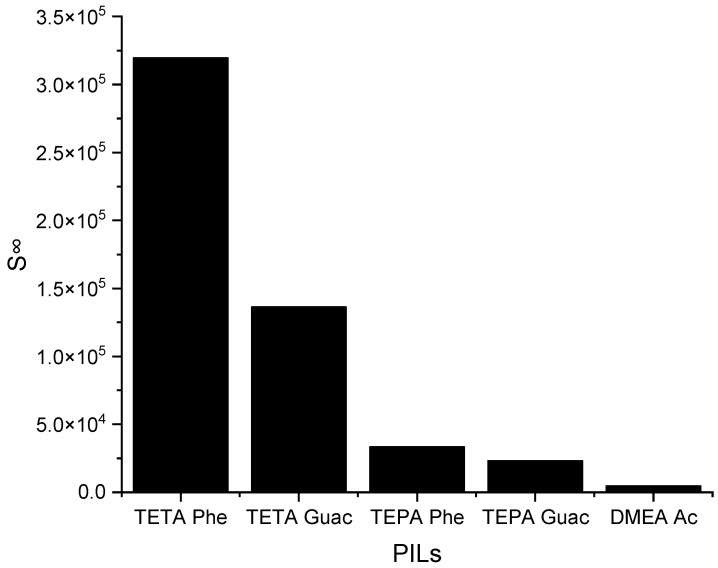
The *S^∞^* of five PILs studied ILs at 298 K.

**Figure 6:**
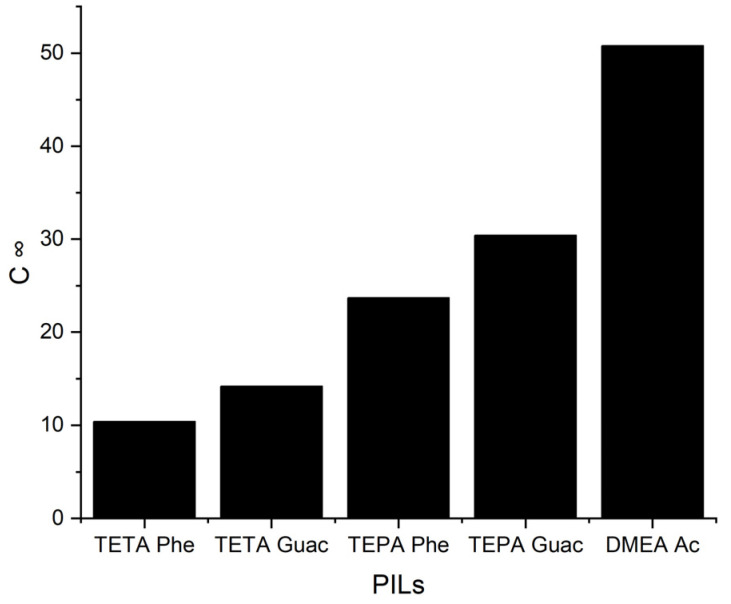
The *C*^∞^ of five PILs at 298 K.

The selected PILs are listed based on the results obtained from the COSMO-RS screening, from largest to smallest. According to these results, all the PILs are capable of forming hydrogen bonds, but [TETA][Phe] exhibits a higher influence on the *S^∞^* compared to the other PILs. The reason behind this is due to the additional effect of its anion, which can contribute to selectivity through π-π interactions. Another factor is its cation structure that influences the ionization of the PIL. Primary amines, like TETA, tend to have higher proton transfer tendencies compared to DMEA, which has a tertiary amine structure, reducing the ionization of the PIL and, thus, providing two different hydrogen bond environments. Additionally, an increase in the alkyl chain length of the cation results in a reduction in ionization, as depicted in Figure 5, regarding TETA and TEPA. Figure 6 below illustrates the *C^∞^* of the five PILs.

**Figure 7:**
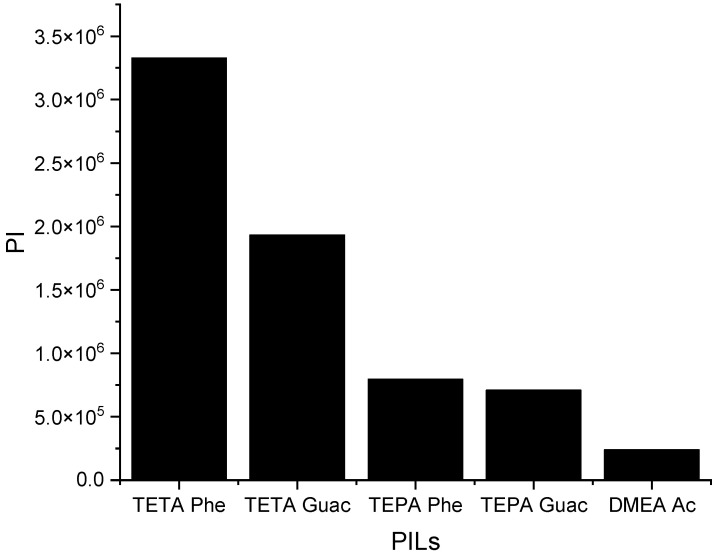
The performance index of PILs at 298 K.

It can be observed from Figure 6 that when the *S^∞^* increases, the *C^∞^* decreases and vice versa. This trend is similar to that of the APILs. It shows that PILs that encompass an aromatic anion influence the capacity, since it increases the steric hindrance to accommodate pyrrole; this contrasts with DMEA that contains a normal acetate anion. In addition, the longer alkyl chain of cations like TEPA weakens the columbic force and leads to a loosening of the stacking structure, which is able to accommodate the pyrrole compound. Finally, the performance index (PI) analysis demonstrates that [TETA][Phe] is the most favorable PIL in terms of both selectivity and capacity, as depicted in Figure 7. However, for industrial utilization, it is crucial to consider criteria such as cost-effectiveness and low toxicity. Guaiacol, a natural and nontoxic compound, serves as an exemplary candidate for the synthesis of anions in PILs. Hence, this screening confirms the capability of PILs as solvents for extracting *N*-containing compounds.

#### 2.1.5. Sigma Profile Analysis

**Figure 8:**
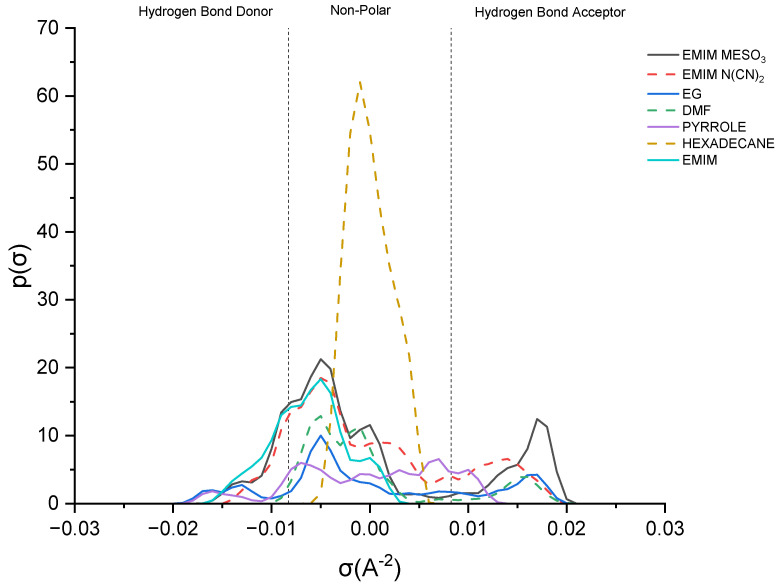
Sigma profile of industrial solvents and ILs.

This study delves into the influence of cation and anion structures on the extractability and interactions between ionic liquids (ILs) and solutes, employing the σ-profile approach. This approach provides insights into how the molecular structures of ILs affect their interactions with solutes. According to the COSMO-RS theory, the charge density of a molecule can be divided into three regions: the hydrogen bond donor region (HBD) (σ < −0.0084 e.Å^−2^), the nonpolar region (−0.0084 e.Å^−2^ < σ < 0.0084 e.Å^−2^), and the hydrogen bond acceptor region (HBA) (σ > 0.0084 e.Å^−2^). These regions serve as the fingerprint features of a molecule [37]. Figure 8 illustrates the sigma profile of both the industrial solvents and ILs. 

As depicted in Figure 8, pyrrole demonstrates a small distribution curve in both the hydrogen bond donor (HBD) and hydrogen bond acceptor (HBA) regions. Therefore, an effective extracting solvent should possess both HBA and HBD characteristics in order to establish favorable interactions with pyrrole. [EMIM]^+^ exhibits a peak in the negative direction, signifying the positive charge on the nitrogen atom [38]. The extent of the interaction between the cations and pyrrole depends on the presence of accessible hydrogen bond donors or acceptors. Although the HBA region for [EMIM]^+^ is non-existent, a weak hydrogen bonding interaction with pyrrole can still occur, due to a portion of the [EMIM]^+^ profile being to the left of the cut-off zone.

The sigma profile of hexadecane falls within the non-polar region, since it consists solely of carbon and hydrogen atoms. Hydrocarbons, being composed of elements with similar electronegativities, exhibit a relatively uniform distribution of electron density, resulting in weak or non-existent interactions with pyrrole. Moving on to the ion pairs, [EMIM][N(CN)_2_] and [EMIM][MeSO_3_], both demonstrate a distribution curve in both the HBD and HBA regions. However, there is a slight dominance in the HBA region, primarily due to the inherent negative charge of the anions [22].

[EMIM][MeSO_3_] exhibits a higher polarization, with a charge density ranging up to 0.021 σ (e/Å^2^), compared to that of [EMIM][N(CN)_2_], with 0.019 σ (e/Å^2^). Additionally, [EMIM][MeSO_3_] provides a larger area for interaction with pyrrole, resulting in a higher capacity. Despite pyrrole exhibiting weak polarization, it can still form weak hydrogen bonding interactions with both ion pairs.

Lastly, focusing on the industrial solvents, EG (ethylene glycol) demonstrates an almost symmetrical sigma profile in both the HBA and HBD regions, whereas DMF (dimethylformamide) exhibits an asymmetrical sigma profile. Nevertheless, both solvents complement pyrrole in forming mutual interactions.

#### 2.1.6. Sigma Potential Analysis

**Figure 9:**
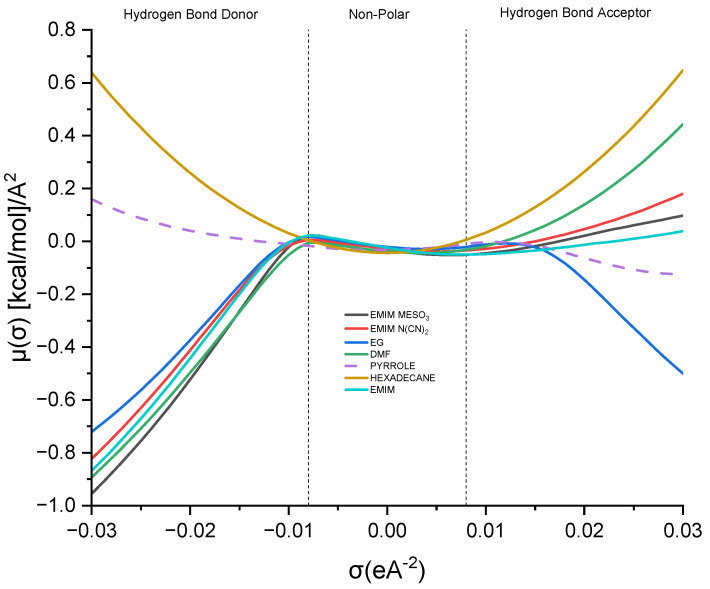
Sigma potential of ion pairs and industrial solvents.

The sigma potential (σ) provides insights into the affinity between solvents in a mixture. A greater negative value of μ (σ) signifies a stronger attraction between molecules, whereas a higher positive value suggests repulsive behavior [39]. Examining Figure 9, the σ-potential curve for hexadecane resembles a parabola, while that for pyrrole demonstrates almost symmetrical characteristics, with a slight affinity towards the hydrogen bond acceptor region.

Analyzing the σ-potential of the [EMIM]^+^ cation reveals a similar pattern to that of the σ-profile, where [EMIM]^+^ exhibits a high affinity as a hydrogen bond donor. Moving on to the other ionic liquids, such as [EMIM][MeSO_3_] and [EMIM][N(CN_2_)], they predominantly occupy the hydrogen bond donor region. This differs slightly from their σ-profile analysis, where both solvents display a dominant presence in the hydrogen bond acceptor region. Nonetheless, these solvents possess high negative values, indicating a complementary interaction with the solute.

Considering the conventional industrial solvents, EG and DMF, it can be observed that EG exhibits almost symmetrical curves in both regions, with a slight dominance in the hydrogen bond donor area. On the other hand, DMF deviates slightly from the σ-profile analysis, tending towards the hydrogen bond donor region. This suggests that DMF has the potential to donate a hydrogen bond to the pyrrole nitrogen atom, facilitating a hydrogen bond interaction between the two molecules.

### 2.2. Experimental Validation

#### 2.2.1. Ternary Liquid–Liquid Equilibrium

**Figure 10:**
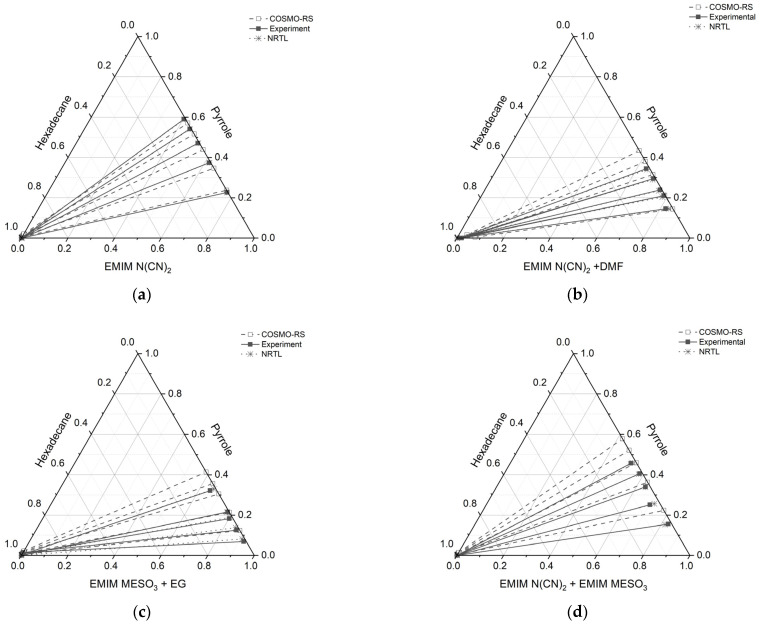
Tie lines for the ternary systems: (**a**) [EMIM][N(CN)_2_] (1) + pyrrole (2) + *n*-hexadecane (3), (**b**) [EMIM][N(CN)_2_] + DMF (1) + pyrrole (2) + *n*-hexadecane (3), (**c**) [EMIM][MeSO_3_] (1) + EG (1) + pyrrole (2) + *n*-hexadecane (3), and (**d**) [EMIM][N(CN)_2_] (1) + [EMIM][MeSO_3_] (1) + pyrrole (2) + *n*-hexadecane (3), at T = 298.15 K and 1 atm.

The molar composition of the tie line for each ternary system, along with the distribution ratio (*D*), selectivity (*S*), and extraction efficiency (E), are tabulated in Table 5. The corresponding triangular phase diagrams are presented in Figure 10.

All the ternary systems investigated in this study exhibited positive slopes, indicating that pyrrole in the extract phase demonstrates a higher affinity for the IL and binary mixture. This characteristic is advantageous for an extracting solvent as it requires a smaller amount of solvent to effectively remove the nitrogen compounds. Comparing the slope of the tie lines in systems containing a binary mixture with a conventional solvent, (such as ([EMIM][N(CN)_2_] + DMF) and ([EMIM][MeSO_3_] + EG)), to systems consisting solely of ILs (([EMIM][N(CN)_2_]) and an IL-IL mixture ([EMIM][N(CN)_2_] + [EMIM][MeSO_3_])), it was observed that the slopes were lower for the former cases. This indicates that the presence of additional pyrrole compound reduces the capacity of ILs with a conventional solvent.

Furthermore, the gradient of the tie lines in Figure 10 for all the systems initially exhibited low values, but increased with an increase in pyrrole concentration. The gradient of the tie line serves as an indicator of the selectivity of the extraction process, and it is closely linked to the theoretical number of extraction steps. Therefore, using a binary mixture of an ionic liquid (IL) with a conventional solvent for the extraction of basic nitrogen compounds leads to good selectivity, but is achieved at the expense of lower capacity. On the other hand, the IL-IL mixture demonstrates favorable outcomes in terms of both capacity and selectivity.

#### 2.2.2. Distribution Ratio and Selectivity

Table 5 presents the data on the distribution ratio and selectivity, showcasing the variations in the concentration of pyrrole in the raffinate phase. The distribution ratio reflects the capacity of the ionic liquids (ILs) and binary mixtures to extract nitrogen compounds from hexadecane. On the other hand, selectivity measures the efficiency of extracting nitrogen molecules from hexadecane while retaining the hexadecane itself. The values in Table 5 show that selectivity is higher across all the systems compared to the distribution ratio. This indicates the selective partitioning of pyrrole into one phase, enhancing the separation between the extract and raffinate phases, resulting in a more efficient extraction process. Additionally, the phase with the higher selectivity value will contain a higher solute concentration, leading to increased purity. This is advantageous when the objective is to extract a specific component from a mixture while minimizing impurities in the final product.

Furthermore, the selectivity observed in [EMIM][N(CN)_2_] and [EMIM][N(CN)_2_] + [EMIM][MeSO_3_] is higher compared to IL + conventional solvents, mainly due to the synergistic effects exhibited by both ILs with pyrrole. Considering the relatively high cost and complex synthesis procedures associated with ILs in comparison to IL + conventional solvents, it becomes evident that IL + conventional solvent mixtures have the potential to serve as viable alternatives, especially for industrial-scale applications. Furthermore, the mole fractions of *n*-hexadecane in the extract phase for all the ternary systems are below 0.01, suggesting minimal cross-contamination between the extract and raffinate phases. This aspect is highly beneficial for separation processes.

#### 2.2.3. Extraction Efficiency

**Figure 11:**
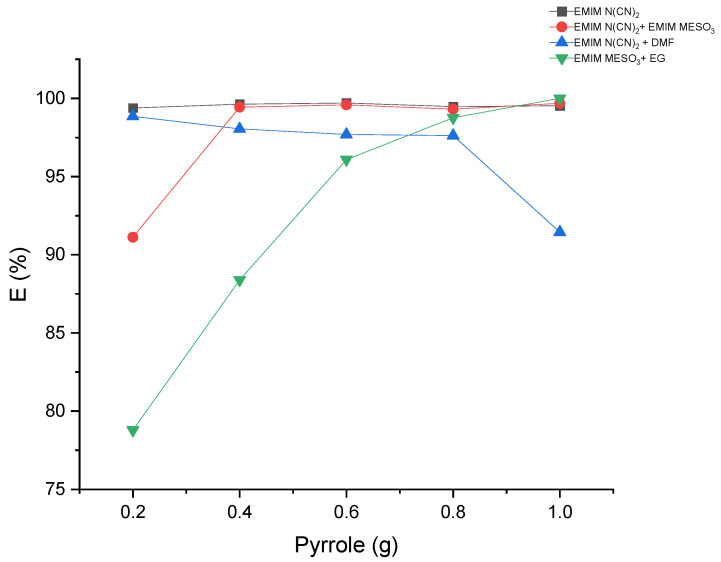
The effect of mass feed of pyrrole on the extraction efficiency.

The extraction of pyrrole from the hexadecane-rich phase was performed using liquid–liquid extraction with various systems comprising ILs and binary mixtures. The results, as shown in Table 5 and Figure 11, demonstrate extraction efficiencies exceeding 78% for all the systems. Interestingly, the system containing [EMIM][N(CN)_2_] + DMF exhibited a decrease in extraction efficiency with an increasing pyrrole concentration. In contrast, the systems [EMIM][MeSO_3_] + EG and [EMIM][N(CN)_2_] + [EMIM][MeSO_3_] displayed an increase in extraction efficiency with higher pyrrole concentrations. This suggests that these solvents may exhibit improved solubility for the solute at higher concentrations, or an enhanced interaction between the solvent and solute. The synergistic effects of ILs in these systems could positively influence the extraction efficiency.

Furthermore, the system [EMIM][N(CN)_2_] demonstrated a steady-state extraction efficiency of 99% as the pyrrole concentration increased. Overall, these findings indicate that the use of ILs and binary mixtures can effectively extract pyrrole, allowing for process optimization and cost-effective extraction processes.

### 2.3. COSMO-RS Prediction vs. Experimental Data

The results obtained from Figure 10 reveal a satisfactory agreement between the COSMO-RS predictions and the experimental data for the system [EMIM][N(CN)_2_], particularly for the raffinate and extract phases, which are rich in hexadecane and IL solvent, respectively. However, for three systems ([EMIM][N(CN)_2_] + DMF, [EMIM][MeSO_3_] + EG, and [EMIM][N(CN)_2_] + [EMIM][MeSO_3_]), slight discrepancies were observed. In these systems, the experimental tie lines exhibited a small amount of hexadecane, which was not predicted by the COSMO-RS model. Conversely, in the raffinate phase of the [EMIM][N(CN)_2_] + DMF system, the COSMO-RS predicted a small amount of pyrrole that was not observed experimentally. It should be noted that the quantities of hexadecane in all the systems were less than 0.01 mole, as shown in Table 5.

In the case of the [EMIM][MeSO_3_] + EG system, at the lowest feed concentration of pyrrole, the experimental tie line displayed a lower slope and gradient compared to the COSMO-RS predictions. Additionally, this system exhibited a relatively high discrepancy between the COSMO-RS and experimental values. However, the positive slope observed in the tie lines indicates that [EMIM][MeSO_3_] + EG has an affinity for pyrrole.

The disparities observed between the COSMO-RS predictions and the experimental data imply the existence of additional factors or complexities that could potentially influence the extraction behavior within these systems. Further investigation and refinement of the model may be necessary to improve the accuracy of the predictions and to better capture the experimental observations.

### 2.4. NRTL Modeling

The LLE phase compositions were determined by solving an isothermal liquid–liquid flash at a specified temperature and pressure, involving the following system of equations:

(1) $${Material\;Balance:\;x}_{i}-\left(1-\omega \right)x_{i}^{L1}-\omega x_{i}^{L2}=0,\;i=1,N_{c}$$

(2) $${Equilibrium\;Equation:\;x}_{i}^{L1}\gamma_{i}^{L1}-x_{i}^{L2}\gamma_{i}^{L2}=0,\;i=1,N_{c}$$

(3) $$Equation\;of\;Summation:\;\sum_{i}x_{i}^{L1}-\sum_{i}x_{i}^{L2}=0$$

In the given equations, ω represents the liquid–liquid splitting ratio, *x_i_* is the quantity of component *i* in the mixture, $x_{i}^{L1}$ denotes the quantity of component *i* in the liquid phase *L*1; $x_{i}^{L2}$ represents the quantity of component *i* in the liquid phase *L*2, and *N_C_* is the number of constituents in the liquid phases. The parameters $\gamma_{i}^{L1}$ and $\gamma_{i}^{L2}$ signify the activity coefficients of component *i* in *L*1 and *L*2, respectively.

In this study, the activity coefficients were computed using the non-random two-liquid (NRTL) model. For a system with multiple components, the activity coefficient of component *i* is determined using the following general expression:

(4) $$\ln\gamma_{i}=\frac{\sum_{j}\tau_{ji}G_{ji}x_{j}}{\sum_{j}G_{ji}x_{j}}+\sum_{j}\frac{G_{ji}x_{j}}{\sum_{k}G_{kj}x_{k}}\left(\tau_{ij}-\frac{\sum_{k}\tau_{kj}G_{kji}x_{k}}{\sum_{k}G_{kj}x_{k}}\right)\;with\;\ln G_{ij}=-\alpha_{ij}\tau_{ij},\alpha_{ij}=\alpha_{ji}\;and\;\tau_{ii}=0$$

Here, *τ_ij_* and *τ_ji_* represent the binary interaction parameters, and α_ij_ corresponds to the non-randomness parameter. The level of non-randomness in the mixture is characterized by the parameter α_ij_. In numerous prior studies involving ILs, a value of 0.2 for the non-randomness parameter has been proven to yield precise fittings for ternary LLE systems. The same value is retained in this study. The model was constructed in the Simulis^®^ environment [[40] In this environment, the binary interaction parameters *τ_ij_* and *τ_ji_* are determined by minimizing the root-mean-square deviation (RMSD) between the calculated and experimental solubilities of each constituent in each phase, as described by the equation:

(5) $$RMSD\;\left(\%\right)=100\sqrt{\sum_{k=1}^{m}\sum_{i=1}^{c}\sum_{j=1}^{2}\frac{{\left(x_{ik}^{j}-{\hat{x}}_{ik}^{j}\right)}^{2}}{2mc}}$$

Here, *x* represents the concentration of a species in mole fraction. The subscripts *i, j,* and *k* denote the component, phase, and tie line, respectively. *m* is the number of tie lines, *c* is the number of components, and *j* is the number of phases, which is two in this case.

The respective RMSD values that correlate with each system are presented in Table 6. The RMSD values for the NRTL correlation consistently fell below 1%, suggesting that the NRTL correlation effectively represents the experimental data. This is further evident in the ternary diagrams, where the tie lines calculated using the NRTL model closely coincide with the experimental tie lines, indicating a high level of agreement between the calculated and experimental data. The NRTL binary interaction parameters regressed for each ternary system are provided in Table 7. To ensure coherence and consistency with our prior findings, the binary interaction parameters between the pyrrole and *n*-hexadecane were adopted from our previous study [27] and remained constant across all the ternary systems, regardless of the IL or IL mixture utilized.

## 3. Materials and Methods

### 3.1. Computational Method

**Figure 12:**
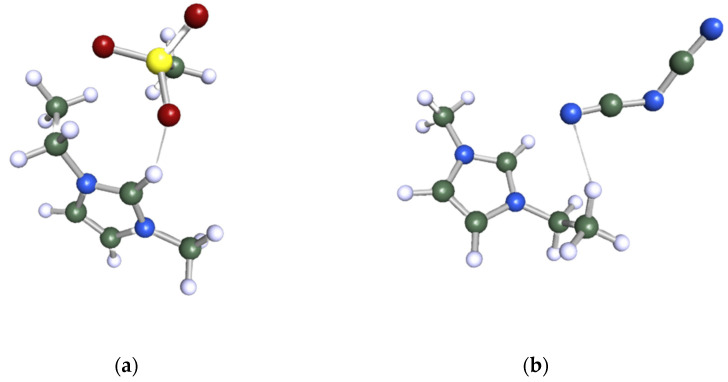
Ball and stick structure of the selected ILs after geometry optimization: (**a**) 1-ethyl-3-methylimidazolium methanesulfonate ([(EMIM][MeSO_3_]) and (**b**) 1-ethyl-3-methylimidazolium dicyanamide ([EMIM][N(CN)_2_]).

The Turbomole program package, version TmoleX 4.0, was employed to carry out the geometry optimization of all the species investigated in this study. Initially, the chemical structure of the target molecule was sketched, followed by a meticulous optimization of its geometry at the Hartree–Fock level using a 6-31G* basis set. To employ the COSMO-RS as a screening tool, it was indispensable to create *.cosmo* files for all the components involved. The *.cosmo* file for each molecule provides comprehensive data regarding the screening charge density (sigma) of the divided molecule within a simulated conductor-like environment. The generation of the .*cosmo* files necessitated a single-point calculation, utilizing density functional theory (DFT) with the Becke–Perdew functional and a Triple-Zeta ζ Valence Potential (TZVP) basis set. Lastly, the COSMOtherm program was employed to export the .*cosmo* files with parameterization BP_TZVP_C30_1401. Figure 12 represents the ball and stick structure of the selected ILs after geometry optimization: (a) 1-ethyl-3-methylimidazolium methanesulfonate ([EMIM][MeSO_3_]) and (b) 1-ethyl-3-methylimidazolium dicyanamide ([EMIM][N(CN)_2_]).

The electroneutral approach was adopted in this work, whereby the complete dissociation of each IL into its constituent cation and anion was assumed. The sigma profile of each IL was obtained by adding the sigma profiles of its constituent cation and anion linearly.

The screened ILs consisted of a total of 13 cations and 31 anions, as reported in Table 8 and Table 9. Various types of cation families were considered, including imidazolium, pyridinium, piperidinium, pyrrolidinium, morpholinium, and pyrazolium. This investigation enables us to observe the effect of the cation type, the impact of increasing the alkyl chain length, and the influence of different anionic species.

The methodology and fundamental equations required to obtain the activity coefficient at infinite dilution have been described comprehensively by Klamt [23]. The infinite dilution activity coefficients (*γ*^∞^) were calculated using COSMOtherm. The capacity (*C^∞^)* and selectivity (*S*^∞^) of the ILs were calculated as follows.

(6) $$C^{\infty }=\frac{1}{\gamma^{\infty }}$$

(7) $$S^{\infty }=\frac{\gamma_{2}^{\infty }}{\gamma_{1}^{\infty }}$$

where $\gamma_{1}^{\infty }$ and $\gamma_{2}^{\infty }$ represent the activity coefficients of pyrrole and hexadecane, respectively. For any solvent, the performance index (*PI*) is calculated as the product of selectivity at infinite dilution and capacity at infinite dilution:

(8) $$PI=C^{\infty }\times S^{\infty }$$

### 3.2. Experimental Methodology

The chemicals utilized in this study are outlined in Table 10 and were utilized in their original state without undergoing additional purification. To ascertain the ternary composition of the extract and the raffinate phases at equilibrium, Nuclear Magnetic Resonance (NMR) spectroscopy was employed with deuterated chloroform (≥99.8%) as the solvent.

#### 3.2.1. LLE Experiment

The feed mixture was assembled within a 20 mL screw-capped scintillation vial, utilizing an analytical balance with a precision of ±0.0001 g. The feed mixture had 10, 20, 30, 40, and 50 wt % pyrrole in hexadecane. The total weight of the feed mixture was standardized to 2 g, following the binary composition, and the ionic liquid (IL) was introduced into each vial at a 1:1 mass ratio. The vials were securely sealed with parafilm to mitigate any loss of the components through evaporation, and spring clamps were used to secure them during shaking. The vials were shaken at 200 rpm in an incubation shaker set at 298.15 K 1 atm for 6 h. Once 6 h had elapsed, the mixing procedure was halted, and the mixture was left untouched for 12 h to achieve equilibrium. This duration was ascertained to be satisfactory following a settling time study, ensuring that the system had indeed reached a state of full equilibrium.

#### 3.2.2. Composition Analysis

To analyze the composition of the extract and raffinate layers, a small volume of the sample (approximately 0.035 mL) was extracted using a micropipette, and precautions were taken to avoid contamination between the layers. A deuterated solvent (CDCl_3_) was added to the NMR tube, and the sample drop was dissolved by gently shaking the tube to create a homogeneous mixture. All the sample systems were dissolved in deuterated chloroform to ensure consistency. The NMR tube was tightly sealed with parafilm to prevent chemical loss during analysis. The 1H NMR spectra of each component were obtained using an NMR 400 MHz Bruker spectrometer, and specific hydrogen peaks with chemical shifts in ppm were selected (EMIM ± 1.6 (3H), MeSO_3_ ± 2.8 (3H), pyrrole ± 6.7 (2H), hexadecane ± 1.27 (28H), DMF ± 2.9 (3H), EG ± 3.7 (4H)) to determine the ternary composition. The molar fraction of each element in both layers was calculated using Equation (9):

(9) $$x_{i}=\frac{H_{i}}{\sum_{i=1}^{3}H_{i}}$$

where $H_{i}$ is the hydrogen peak area in component ⅈ and $x_{i}$ is the mole fraction of species ⅈ. The average uncertainty in the mole fraction for all the experiments was approximated to be less than 0.003.

#### 3.2.3. Experimental Selectivity and Distribution Ratio

Equations (10) and (11) were utilized to assess the extraction effectiveness using the distribution ratio ${(D}_{p})$ and the solvent selectivity (*S*) of each IL.

(10) $$D_{p}=\frac{x_{p}^{1}}{x_{p}^{2}}$$

(11) $$S=\frac{x_{p}^{1}}{x_{p}^{2}}∕\frac{x_{H}^{1}}{x_{H}^{2}}$$

where the concentrations of pyrrole and hexadecane, respectively, are represented by $x_{p}$ and $x_{H}.$ The extract and raffinate phase are denoted by the superscripts 1 and 2, respectively.

#### 3.2.4. Extraction Efficiency

The extraction efficiency was calculated using Equation (12):

(12) $$E=\left(1-\frac{c_{w_{0}}}{c_{w}}\right)\times 100$$

where *C_wo_* and *C_w_* are the concentration of pyrrole in the raffinate and extract phase, respectively.

## 4. Conclusions

In this research, a comprehensive screening involving 173 potential ILs was conducted using the COSMO-RS model. Subsequently, the top two ILs identified were subjected to experimental denitrogenation testing. The utilization of IL-IL mixtures demonstrated a promising capacity and selectivity for extracting nitrogen compounds. Conversely, employing a binary mixture of an IL with a conventional solvent yielded high selectivity but limited capacity. Notably, the positive slope observed for the tie lines across all the systems signifies the strong affinity of nitrogen compounds for ILs and binary mixtures, making them efficient with a reduced amount of solvent required for extraction. Furthermore, a successful tie line correlation was achieved in all the ternary diagrams using the NRTL model. Overall, this study presents a novel and potentially cost-effective approach for denitrogenating fuel oil employing ILs.

## Figures and Tables

**Table 1:** Top 10 ILs based on performance index.

Ionic Liquids	*C^∞^*	*S^∞^*
[EMMOR][PF_6_]	1.15	111,700,401.70
[TMPYZO][BF_4_]	2.14	20,137,639.62
[EPY][PF_6_]	1.83	17,787,295.95
[TMPYZO][PF_6_]	1.39	13,245,829.55
[EMIM][PF_6_]	1.97	6,179,394.27
[MIMPS][H_2_PO_4_]	3.72	3,585,065.70
[TMPYZO][SNC]	2.93	3,227,154.18
Sulfonium [N(CN_)2_]	6.05	2,945,169.56
[EPY][BF_4_]	4.88	2,850,423.02
[EMMOR][triflate]	3.51	2,203,351.52

**Table 2:** Value of selected cation for alkyl chain effect based on *C*^∞^ and *S^∞^*.

Ionic Liquids	*C^∞^*	*S^∞^*
[C_10_MIM][EtSO_4_]	13.19	2006.05
[C_7_MIM][EtSO_4_]	13.02	5234.46
[C_5_MIM][EtSO_4_]	8.88	21,461.10
[C_2_MIM][EtSO_4_]	10.14	76,244.02
[BZMIM][EtSO_4_]	6.79	141,153.68

**Table 3:** Selectivity at infinite dilution (*S^∞^)* based on imidazolium cation with different anions.

Ionic Liquid	*S^∞^*	*C^∞^*
[EMIM][PF_6_]	6,179,394.27	1.97
[EMIM][SNC]	187,565.78	8.28
[EMIM][C_7_H_7_O_3_S]	40,044.79	15.29
[EMIM][C_10_H_19_O_2_]	4682.50	35.91
[EMIM][Br]	214.20	69.55

**Table 4:** The effect of different types of cations on *S*^∞^ and *C^∞^*.

ILs	*C^∞^*	*S^∞^*
[EPYRO][Br]	179.81	12.48
[EMMOR][Br]	135.07	12.88
[EPIP][Br]	127.58	48.29
[EMIM][Br]	69.55	214.2
[EPY][Br]	64.33	248.71
[TMPYZO][Br]	18.07	10,367.81

**Table 5:** Experimental LLE results for systems containing: [EMIM][N(CN)_2_], (1) + pyrrole (2) + *n*-hexadecane (3); [EMIM][N(CN)_2_], + DMF (1) + pyrrole (2) + *n*-hexadecane (3); [EMIM][MeSO_3_] (1) + EG (1) + pyrrole (2) + *n*-hexadecane (3); and [EMIM][N(CN)_2_] (1) + [EMIM][MeSO_3_] (1) + pyrrole (2) + *n*-hexadecane (3), at T = 298.15 K and P = 1 atm.

IL-Rich Phase			Hydrocarbon-Rich Phase					
[EMIM][N(CN)_2_] (1) + Pyrrole (2) + *n*-Hexadecane (3)						*D*	*S*	*E %*
X′1	X′2	X′3	X″1	X″2	X″3			
0.770	0.227	0.003	0	0.001	0.986	162.23	53,437.50	99.4
0.619	0.374	0.007	0	0.001	0.999	267.34	36,825.40	99.6
0.522	0.471	0.007	0	0.001	0.999	336.77	49,656.86	99.7
0.452	0.541	0.006	0	0.003	0.997	193.86	30,372.67	99.5
0.403	0.590	0.007	0	0.001	0.999	211.31	29,243.24	99.5
[EMIM][N(CN)_2_] (1) + DMF (1) + Pyrrole (2) + *n*-Hexadecane (3)								
0.752	0.239	0.009	0.024	0.003	0.974	87.78	10,000.00	98.9
0.782	0.210	0.007	0.019	0.004	0.977	51.18	7246.38	98.1
0.696	0.295	0.009	0.019	0.007	0.974	43.28	4699.09	97.7
0.640	0.344	0.016	0.019	0.008	0.974	42.05	2576.99	97.6
0.822	0.146	0.031	0.018	0.012	0.988	11.69	369.22	91.5
[EMIM][MeSO_3_] (1) + EG (1) + Pyrrole (2) + *n*-Hexadecane (3)								
0.920	0.069	0.011	0	0.015	0.985	4.715	423.98	78.8
0.861	0.125	0.014	0	0.015	0.985	8.614	625.12	88.4
0.801	0.182	0.017	0	0.007	0.993	25.57	1536.10	96.1
0.650	0.322	0.029	0	0.004	0.996	80.82	2797.87	98.8
0.778	0.216	0.006	0	0.000	1.000	(-)	(-)	100.0
[EMIM][N(CN)]2 (1) + [EMIM][MeSO_3_] (1) Pyrrole (2) + *n*-Hexadecane (3)								
0.829	0.156	0.015	0	0.014	0.986	11.26	732.92	91.1
0.703	0.251	0.046	0	0.001	0.999	179.53	3879.31	99.4
0.639	0.340	0.021	0	0.001	0.999	243.18	11,754.39	99.6
0.581	0.406	0.013	0	0.003	0.997	145.51	11,391.75	99.3
0.519	0.457	0.025	0	0.001	0.999	326.71	13,192.49	99.7

**Table 6:** RMSD values for NRTL correlation of ternary LLE systems.

Ternary System	RMSD %
[EMIM][N(CN)_2_] (1) + pyrrole (2) + *n*-hexadecane (3)	0.161
[EMIM][N(CN)_2_]/DMF (1) + pyrrole (2) + *n*-hexadecane (3)	0.785
[EMIM][MeSO_3_]/EG (1) + pyrrole (2) + *n*-hexadecane (3)	0.660
[EMIM][N(CN)_2_]/[EMIM][MeSO_3_] (1) + pyrrole (2) + *n*-hexadecane (3)	0.862

**Table 7:** The values of NRTL binary interaction parameters for each ternary system.

*i* − *j*	$\tau_{ij}$	$\tau_{ji}$
pyrrole—[EMIM][N(CN)_2_]	941.9	−1164.6
pyrrole—[EMIM][N(CN)_2_]/DMF	913.8	−1190.2
pyrrole—[EMIM][MeSO_3_]/EG	855.0	−1095.0
pyrrole—[EMIM][N(CN)_2_]/[EMIM][MeSO_3_]	908.0	−1193.4
*n*-hexadecane—[EMIM][N(CN)_2_]	2576.8	1124.5
*n*-hexadecane—[EMIM][N(CN)_2_]/DMF	2631.7	735.8
*n*-hexadecane—[EMIM][MeSO_3_]/EG	2541.0	892.0
*n*-hexadecane—[EMIM][N(CN)_2_]/[EMIM][MeSO_3_]	2652.0	579.4
pyrrole—*n*-hexadecane	1073.9	824.7

**Table 8:** List of cations for the screened ILs.

No.	Cation Name	Abbreviation
1	1-alkyl-3-methylimidazolium	CnMim+; R = 2, 4, 6, 8
2	1-alkyl-1-methylpyrrolidinium	CnMpyrro+; R = 2, 4, 6, 8
3	1-Ethyl-1-methylpiperidinium	EPIP
4	1-Ethylpyridinium	EPY
5	4-Ethyl-4-methylmorpholinium	EMMOR
6	1,2,4 Trimethylpyrazolium	TMPYZO
7	Cyclic ethylated tetrahydrothiophenium dicyanamide	S2
8	1-Hexyl-3,5-dimethylpyridinium	C6mmPy
9	1-Benzyl-3-methylimidazolium	Bzmim
10	Triethylammonium	TEA
11	Triethylenetetramine	TETA
12	Tetraethylenepentamine	TEPA
13	Dimethylethanolamine	DMEA

**Table 9:** List of anions for the screened ILs.

No.	Anion Name	Abbreviation
1	Bromide	Br
2	Nitrate	NO_3_^−^
3	Thiocyanate	SCN^−^
4	Acetate	CH3COO^−^
5	Bisulfate	HSO_4_^−^
6	Tetrafluoroborate	BF_4_^−^
7	Methylsufonate	CH_3_SO_3_^−^
8	Triflouroacetate	CF_3_COO^−^
9	Methyl sulfate	CH_3_SO_4_^−^
10	Hexafluorophosphate	PF_6_^−^
11	Triflate	CF_3_SO_3_^−^
12	Ethyl Sulfate	C_2_H_5_SO_4_^−^
13	Dimethylphosphate	(CH_3_)_2_PO_4_^−^
14	Methylsulfonylacetamide	C_3_H_7_NO_3_S^−^
15	Tetracyanoborate	C_4_BN_4_^−^
16	Salicylate	C_7_H_5_O_3_^−^
17	Bismethylsulfonylamide	C_2_H_6_NO_4_S_2_^−^
18	Bioxaloborate	BH_2_O_4_^−^
19	Diethylphosphate	C_4_H_11_O_4_P^−^
20	Tosylate	C_7_H_7_O_3_S^−^
21	Trifluoromethanesulfinate	CF_3_SO_2_^−^
22	bis(trifluoromethanesulfonyl)imide	Tf_2_N^−^
23	2-(2-Methoxyethoxy) ethylsulfate	C_5_H_11_O_6_S^−^
24	Decanoate	C_10_H_19_O_2_^−^
25	Octylsulfate	C_8_H_17_O_4_S^−^
26	Dicyanamide	[N(CN)_2_]^−^
27	Benzoate	BzO^−^
28	Hydrogensulfate	HSO_4_^−^
29	Dihydrogen phosphate	H_2_PO_4_^−^
30	Phenolate	C_6_H_5_O^−^
31	Guaiacol	C_7_H7O_2_^−^

**Table 10:** Chemicals used in the LLE experiment.

Chemical Name	CAS No.	Supplier	Purity (wt%)	Abbreviation
1-ethyl-3-methylimidazolium Methanesulfonate	145022-45-3	Sigma Aldrich, Darmstadt, Germany	≥95	[EMIM][MeSO_3_]
1-ethyl-3-methylimidazolium dicyanamide	370865-89-7	Sigma Aldrich, Darmstadt, Germany	≥98.0	[EMIM][N(CN)_2_]
Ethylene Glycol	107-21-1	Merck, Darmstadt, Germany	≥99.0	EG
*N*,*N*-Dimethylformamide	68-12-2	Merck, Darmstadt, Germany	≥99.8	DMF
Pyrrole	109-97-7	Sigma Aldrich, Darmstadt, Germany	99.0	Pyr
Hexadecane	544-76-3	Sigma Aldrich, Darmstadt, Germany	99.0	Hexa
Chloroform-d	865-49-6	Sigma Aldrich, Darmstadt, Germany	99.8	CDCl_3_

## Data Availability

Data are contained within the article.
